# Pertussis and Influenza Vaccination Among Insured Pregnant Women — Wisconsin, 2013–2014

**Published:** 2015-07-17

**Authors:** Ruth Koepke, Danielle Kahn, Ashley B. Petit, Stephanie L. Schauer, Daniel J. Hopfensperger, James H. Conway, Jeffrey P. Davis

**Affiliations:** 1 Wisconsin Division of Public Health; 2 Department of Pediatrics, School of Medicine and Public Health, University of Wisconsin-Madison

On February 22, 2013, the Advisory Committee on Immunization Practices (ACIP) revised recommendations for vaccination of pregnant women to recommend tetanus-diphtheria-acellular pertussis vaccine (Tdap) during every pregnancy, optimally at 27–36 weeks of gestation, to prevent pertussis among their newborns (1). Since 2004, influenza vaccination has been recommended for pregnant women in any trimester to prevent influenza and associated complications for mother and newborn (2). To evaluate vaccination of pregnant women in Wisconsin after the 2013 Tdap recommendation, health insurance claims data for approximately 49% of Wisconsin births were analyzed. The percentage of women who received Tdap during pregnancy increased from 13.8% of women delivering during January 2013 (63.1% of whom received Tdap 2–13 weeks before delivery) to 51.0% of women delivering during March 2014 (90.9% of whom received Tdap 2–13 weeks before delivery). Among women delivering during November 2013–March 2014, 49.4% had received influenza vaccine during pregnancy. After the 2013 recommendation, Tdap vaccination among pregnant women increased but plateaued at rates similar to influenza vaccination rates. Prenatal care providers should implement, evaluate, and improve Tdap and influenza vaccination programs, and strongly recommend that pregnant patients receive these vaccines to prevent severe illness and complications among mothers and infants.

Infants too young for vaccination have the greatest risk for severe pertussis morbidity and mortality. Tdap vaccination of pregnant women stimulates production of maternal antipertussis antibodies which are transplacentally transported to the fetus, providing passive protection to newborn infants. Results of studies conducted in the United Kingdom indicate that Tdap vaccination during the third trimester is approximately 90% effective in preventing pertussis among infants aged <2 months (3,4). ACIP first recommended Tdap during pregnancy in 2011; women who had previously not received Tdap were recommended to receive it, preferably after 20 weeks of gestation (5). After the 2011 recommendation, Tdap vaccination rates among pregnant women were low (6,7), and results of antibody persistence studies suggested that Tdap vaccination before pregnancy or during early pregnancy might not provide sufficient levels of maternal antibodies to the fetus (8). Therefore, ACIP revised its recommendation to recommend Tdap during every pregnancy. Additionally, because ≥2 weeks are needed after Tdap vaccination for the mother to mount a maximal immune response and antibody transport across the placenta is greatest after 30 weeks of gestation, ACIP recommended Tdap administration to pregnant women at 27–36 weeks of gestation (1).

The Wisconsin Health Information Organization Datamart is a deidentified all-payer claims database that contains a rolling 24 months of medical and pharmacy claims data from Wisconsin Medicaid and most private insurance plans in Wisconsin.* Claims data were extracted from Datamart version 12, which included services during April 2012–March 2014. Pregnant women and their delivery dates were identified using *International Classification of Diseases, Ninth Revision* and *Current Procedural Terminology* (CPT) codes that indicate delivery.† Women aged 11–44 years with deliveries during the January 2013–March 2014 study period were included; each woman was included once. Vaccinations received by these women during April 2012–March 2014 were identified using CPT codes (Tdap, 90715; influenza, 90654–90662, 90672, 90673, 90685–90688, and 90724). Vaccination during the 40 weeks before the delivery date was considered vaccination during pregnancy. Because gestational age data were not available, vaccination 2–13 weeks before delivery was used to evaluate Tdap receipt during the recommended time. Percentages of women who received Tdap, influenza, or both vaccines during pregnancy were calculated by month and year of delivery. During delivery months November 2013–March 2014, an interval during influenza season when vaccination rates were stable, vaccination rates were compared by maternal age, county of residence, delivery provider specialty, and insurance type.

The study population included 40,054 women with deliveries during the study period and represented approximately 49% of deliveries in Wisconsin. Median maternal age was 28 years. Residents of the two most populous counties (Milwaukee and Dane) accounted for 33.9% of the women (Table). Most (75.6%) delivery providers were obstetrician/gynecologists; 65.8% of women were insured by Medicaid.

Among the 40,054 women, 14,033 (35.0%) received Tdap during pregnancy. The percentage of women who received Tdap during pregnancy increased from 13.8% among women delivering during January 2013 to 51.0% among women delivering during March 2014 (Figure 1). Among women who received Tdap during pregnancy, the percentage who received Tdap 2–13 weeks before delivery increased from 63.1% among women delivering during January 2013 to 90.9% among women delivering during March 2014 (Figure 2).

Influenza vaccine was received during pregnancy by 15,501 (38.7%) women. The percentage of women who received influenza vaccine during pregnancy was lowest among women who delivered during July–September 2013 and higher among women who delivered during the 2012–13 and 2013–14 influenza seasons (Figure 1). Among women delivering during November 2013–March 2014, 49.4% received influenza vaccine during pregnancy. Receipt of both Tdap and influenza vaccines during pregnancy increased from 9.3% of women delivering during January 2013 to 34.7% of women delivering during November 2013–March 2014 (Figure 1).

Among 12,089 (30.2%) women delivering during November 2013–March 2014, vaccination rates were highest among women aged 30–34 years and lowest among women aged 11–19 years (Table). Dane County residents had higher vaccination rates than Milwaukee County and other Wisconsin residents. Women delivering to family medicine or general practitioner providers had higher vaccination rates than women delivering to obstetrician/gynecologists or nurse practitioners/midwives. Vaccination rates were higher among women with private insurance than women with Medicaid.

## Discussion

After the February 2013 ACIP recommendation, Tdap vaccination of pregnant women in Wisconsin increased steadily but plateaued near 50% during November 2013–March 2014. During this 5-month period coinciding with the 2013–14 influenza season, a similar percentage of pregnant women were reported to have received influenza vaccine during pregnancy. However, only 34.7% received both vaccines during pregnancy. These findings indicate that despite the rapid implementation of Tdap vaccination among pregnant women in Wisconsin, many pregnant women did not receive both recommended vaccines, including women who demonstrated a willingness to receive at least one other vaccine during pregnancy.

To optimize the concentration of antipertussis antibodies transported across the placenta from mother to infant, ACIP recommends Tdap administration at 27–36 weeks of gestation, during the third trimester and ≥2 weeks before delivery. After the 2013 recommendation, the percentage of women vaccinated 2–13 weeks before delivery increased to 90.9% among Tdap-vaccinated pregnant women who delivered during March 2014. This finding indicates that among women vaccinated with Tdap during pregnancy, Tdap was typically received during the time expected to confer the greatest level of protection to the infant.

This study evaluated implementation of ACIP’s 2013 Tdap recommendation among publicly and privately insured pregnant women across multiple health care providers. Tdap vaccination rates among women who delivered during January 2013 were similar to rates reported in other U.S. states before the February 2013 recommendation (6,7). After the 2013 recommendation, one Massachusetts hospital reported most (81.6%) pregnant patients had received Tdap, but most were vaccinated after 37 weeks of gestation (9). Results of a national Internet panel survey demonstrated that among women pregnant anytime during October 2013–January 2014, 34.6% reported receiving influenza vaccine during pregnancy (10).

Among characteristics examined in this study, Tdap and influenza vaccination rates during pregnancy were lowest among women who were aged <20 years, resided in Milwaukee County, were insured by Medicaid, and delivered to nurse practitioners or midwives, although nurse practitioners and midwives represented <8% of delivery providers. Previous studies of vaccination rates among pregnant women have identified differences by maternal age, race, poverty level, and prenatal care adequacy (6,7,9,10). These differences highlight the importance of public health programs using local data to identify disparities and target interventions to specific populations and health care providers. However, even among women in Wisconsin who delivered to family physicians and general practitioners, less than half had received both Tdap and influenza vaccine, and among those who delivered to obstetricians and gynecologists, only about one third had received both vaccines during November 2013–March 2014.

The findings in this report are subject to at least two limitations. First, only deliveries and vaccinations properly coded, paid by the insurer, and submitted to the Datamart database were included. Therefore, vaccination rates might be underestimated if vaccinations were received but not paid by the insurer, and the findings in this report are not generalizable to uninsured women, women insured by payers not included in the database, or women outside of Wisconsin. Second, because the database did not include gestational age data, neither the exact week of pregnancy during which Tdap was received nor the effect of preterm birth on vaccination during pregnancy could be evaluated.

Health care provider recommendation and offer of vaccination are among the strongest predictors of whether a woman will be vaccinated during pregnancy (10). Health care providers are encouraged to strongly recommend and offer Tdap and influenza vaccination during pregnancy and to use materials developed by CDC§ to educate patients regarding the importance of vaccination during pregnancy to prevent illness and severe complications among mothers and infants.

**Summary**

What is already known on this topic?

Pertussis (whooping cough) incidence is increasing in the United States, including among infants, who are at highest risk for hospitalization and death. To prevent pertussis among newborn infants, pregnant women are recommended to receive tetanus-diphtheria-acellular pertussis vaccine (Tdap) during every pregnancy, a strategy that provides passive protection to the newborn infant. Additionally, pregnant women are recommended to receive influenza vaccine during pregnancy to prevent influenza-associated complications among mothers and infants.

What is added by this report?

After the 2013 Advisory Committee on Immunization Practices guidelines that recommended Tdap vaccination during every pregnancy, Tdap vaccination rates among privately and publicly insured pregnant women in Wisconsin increased quickly but plateaued at rates similar to influenza vaccination rates. Tdap and influenza vaccination rates were lowest among women who were younger, had public insurance, resided in Milwaukee County, and had nurse practitioners or midwives as delivery providers.

What are the implications for public health practice?

Collaboration among public health programs and providers of prenatal care is needed to identify and overcome barriers to improving vaccination rates among pregnant women.

## Figures and Tables

**FIGURE 1:**
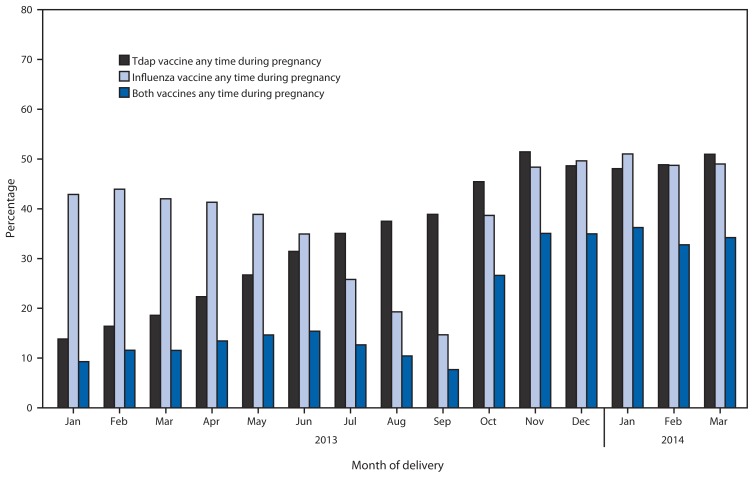
Percentage of the study population who received Tdap, influenza, or both vaccines during pregnancy, by month of delivery — Wisconsin, January 2013–March 2014 **Abbreviation:** Tdap = tetanus-diphtheria-acellular pertussis.

**FIGURE 2:**
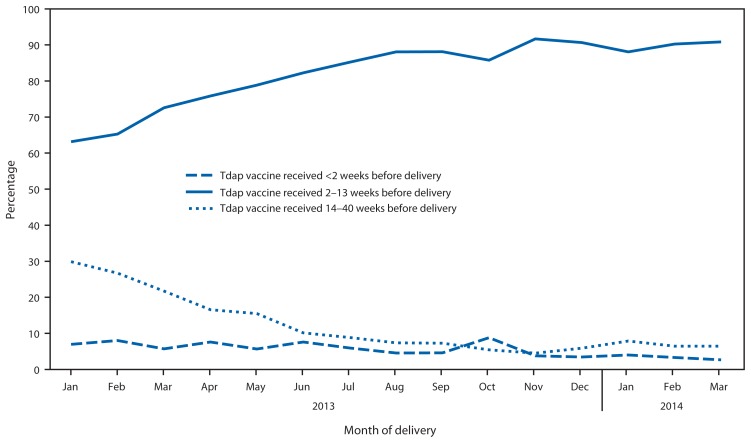
Timing of Tdap vaccine receipt among women in the study population who received Tdap vaccine during pregnancy, by month of delivery — Wisconsin, January 2013–March 2014 **Abbreviation:** Tdap = tetanus-diphtheria-acellular pertussis.

**TABLE:** Percentage of the study population who received Tdap, influenza, or both vaccines during pregnancy, by maternal and health care provider characteristics and delivery period — Wisconsin, January 2013–March 2014

	Delivery period											
	
	January 2013–March 2014				November 2013–March 2014							
		
	Total		Tdap		Total		Tdap		Influenza		Both	
						
Characteristic	No.	(%)	No.	(%)	No.	(%)	No.	(%)	No.	(%)	No.	(%)
**Total study population**	**40,054**	**(100.0)**	**14,033**	**(35.0)**	**12,089**	**(100.0)**	**5,992**	**(49.6)**	**5,970**	**(49.4)**	**4,194**	**(34.7)**
**Maternal age at delivery (yrs)**												
11–19	**2,604**	**(6.5)**	737	(28.3)	**849**	**(7.0)**	352	(41.5)	392	(46.2)	247	(29.1)
20–24	**9,818**	**(24.5)**	3,070	(31.3)	**2,979**	**(24.6)**	1,308	(43.9)	1,394	(46.8)	942	(31.6)
25–29	**12,482**	**(31.2)**	4,454	(35.7)	**3,801**	**(31.4)**	1,969	(51.8)	1,865	(49.1)	1,328	(34.9)
30–34	**10,276**	**(25.7)**	3,951	(38.4)	**3,029**	**(25.1)**	1,650	(54.5)	1,594	(52.6)	1,174	(38.8)
35–39	**4,069**	**(10.2)**	1,538	(37.8)	**1,203**	**(10.0)**	600	(49.9)	616	(51.2)	431	(35.8)
40–44	**805**	**(2.0)**	283	(35.2)	**228**	**(1.9)**	113	(49.6)	109	(47.8)	72	(31.6)
**Maternal county of residence** *												
Dane County	**5,075**	**(12.7)**	2,719	(53.6)	**1,614**	**(13.4)**	1,106	(68.5)	1,036	(64.2)	843	(52.2)
Milwaukee County	**8,477**	**(21.2)**	2,382	(28.1)	**2,423**	**(20.0)**	902	(37.2)	1,017	(42.0)	645	(26.6)
All other Wisconsin counties	**26,502**	**(66.2)**	8,932	(33.7)	**8,052**	**(66.6)**	3,984	(49.5)	3,917	(48.6)	2,706	(33.6)
**Specialty of delivery provider** †												
Family medicine/General practitioner	**5,417**	**(13.5)**	2,202	(40.6)	**1,604**	**(13.3)**	898	(56.0)	928	(57.9)	686	(42.8)
Nurse practitioner/Midwife	**3,150**	**(7.9)**	1,087	(34.5)	**922**	**(7.6)**	403	(43.7)	418	(45.3)	274	(29.7)
Obstetrician/Gynecologist	**30,299**	**(75.6)**	10,396	(34.3)	**9,182**	**(76.0)**	4,522	(49.2)	4,450	(48.5)	3,128	(34.1)
**Type of insurance** §												
Private	**13,617**	**(34.0)**	5,960	(43.8)	**4,194**	**(34.7)**	2,588	(61.7)	2,324	(55.4)	1,779	(42.4)
Medicaid	**26,337**	**(65.8)**	8,029	(30.5)	**7,880**	**(65.2)**	3,394	(43.1)	3,637	(46.2)	2,406	(30.5)

**Abbreviation:** Tdap = tetanus-diphtheria-acellular pertussis.

* U.S. Census Bureau estimate of percentage of population under federal poverty level during 2009–2013: Dane County, 12.9%; Milwaukee County, 21.6%; and Wisconsin, 13.0%.

† Data not shown for 1,188 deliveries with unknown provider specialty.

§ Data not shown for deliveries paid for by Medicare (four), the Federal Employee Program (47), or unknown type of insurance (49).

## Footnotes

* Additional information available at http://wisconsinhealthinfo.org/about.

† Additional information available at http://www.ncqa.org/portals/0/Prenatal%20Postpartum%20Care.pdf.

§ Available at http://www.cdc.gov/pertussis/pregnant/index.html and http://www.cdc.gov/pertussis/materials/pregnant.html.
